# Exploring motor variability in adults with severe athetoid or ataxic cerebral palsy: Impact on error-based and reward-based learning

**DOI:** 10.1371/journal.pone.0349610

**Published:** 2026-06-22

**Authors:** Alba Roldan, María Isabel Cornejo, Francisco J. Moreno, Raul Reina, Carla Caballero

**Affiliations:** 1 Sports Research Centre, Department of Sport Sciences, Miguel Hernández University of Elche, Elche, Spain; 2 Escuela de Kinesiología, Facultad de Salud, Universidad Santo Tomás, Santiago, Chile; 3 Neurosciences Research Group, Alicante Institute for Health and Biomedical Research (ISABIAL), Alicante, Spain; University of Illinois Urbana-Champaign, UNITED STATES OF AMERICA

## Abstract

Individuals with cerebral palsy (CP) are characterized by elevated movement variability. Movement variability has been proposed as a mechanism that facilitates the exploration of diverse movement strategies. However, in the case of CP, this variability is often excessive, potentially impeding effective error correction and motor learning processes. This study aims to investigate motor variability in adults with severe athetoid and ataxic CP profiles, focusing on its impact on error-based and reward-based learning mechanisms. The study included 13 adults with severe CP and a control group of 18 adults without disabilities. The findings revealed that individuals with CP exhibit significantly greater motor variability than the control group, which correlates with higher absolute error rates and a diminished capacity for task-related adjustments. These results suggest that CP negatively affects motor stability and precision, complicating motor learning. Furthermore, it was observed that error-based tasks are more accessible for individuals with CP, as they showed substantial performance improvements following training, in contrast to the reward-based tasks. This study underscores the importance of personalized motor learning interventions for individuals with CP, considering the variability among CP subtypes and the individual characteristics of each person. The research provides essential insights into motor learning adaptation and retention in individuals with severe CP, highlighting the necessity of continuous practice to sustain long-term improvements.

## Introduction

Examining motor variability is crucial for understanding its distinct role in motor learning, since the nature of variability itself is a critical factor in human movement and a key aspect of motor learning [1,2]. However, motor control research often yields controversial results, reflecting the complexity of learning processes, showing that the relationship between variability and learning depends on the task features and the individual's differences during skill acquisition [3,4].

Motor variability refers to the natural fluctuations in performance that occur when individuals repeat a task. It seems that this variability is not merely noise but can facilitate exploring different movement strategies, allowing individuals to adapt and find optimal solutions to motor problems [5]. Some studies have shown that greater intrinsic variability is often associated with faster learning, as it encourages the exploration of the motor system's degrees of freedom [6]. Nevertheless, variability has been examined in relation to the type of task. Research distinguishes between error-based tasks, where individuals learn through correcting mistakes, and reward-based tasks, which focus on positive reinforcement. Each type of task influences the learning process differently, where error-based learning relies on feedback from mistakes to refine movements, while reward-based learning uses positive outcomes to reinforce correct actions [7].

In reward-based learning, higher task-relevant motor variability would predict faster learning, suggesting the crucial role of exploration in discovering effective motor strategies [6,8]. While a higher task-relevant motor variability would facilitate faster learning, as it promotes exploration of different action strategies, an excessive motor variability generally impairs performance in error-based tasks that rely on precise error correction [6,9]. In other words, an excessive variability can interfere with the precision required for effective error correction [9], so the role of variability in this type of task could be more closely tied to the individual's ability to detect and correct errors rather than to exploratory activities. This is highlighted by research showing that lower auto-correlated variability (a measure of structured variability) is linked to higher learning rates due to increased error sensitivity [1]. These differences reflect distinct underlying neural substrates: the cerebellum is central to error-based learning and sensorimotor adaptation driven by sensory prediction errors [10], whereas the basal ganglia support reinforcement- and reward-based motor learning via dopaminergic reward prediction error signaling [11]. Accordingly, cerebellar damage (as observed in ataxia) and basal ganglia dysfunction (as in dystonia/athetosis) would be expected to differentially influence these learning mechanisms.

Hence, the individual's differences are another critical factor that influences the motor variability implications on learning processes. Despite the extensive study of motor variability, its role in individuals with brain damage, such as those with cerebral palsy (CP) or traumatic brain injury, has been less explored. This gap is even more pronounced in populations with severe impairments. CP manifests in various movement disorders, including ataxia and dyskinesia [12], which exhibit distinct motor characteristics. Ataxia is primarily associated with cerebellar dysfunction, resulting in impaired coordination, poor balance, and an unsteady gait. Individuals often experience difficulty in coordinating voluntary movements, resulting in issues such as dysarthria and nystagmus [13]. In contrast, dyskinesia encompasses both dystonia and athetosis [14]. Specifically, dystonia is characterised by involuntary fluctuations in muscle activation patterns during voluntary movements or while maintaining posture; while athetosis involves slow, involuntary writhing movements that predominantly affect the extremities, often resulting from damage to the basal ganglia. These movements are continuous and can significantly interfere with daily activities, causing irregular and unpredictable muscle contractions [14,15]. Hence, while ataxia results in broad coordination deficits affecting balance and speech, athetosis is characterized by involuntary and fluctuating movements that impair motor control and posture. These alterations may compromise the ability to detect and correct errors during movement execution, a process that is crucial for the acquisition and refinement of motor skills [16].

Understanding motor variability in CP is crucial for effective therapeutic interventions. Research has shown that individuals with CP exhibit greater movement variability in motor skill learning compared to their non-disabled peers [11]. Additionally, their motor learning is influenced not only by intrinsic variability but also by task constraints. For instance, adults with ataxia demonstrate lower sensorimotor adaptation in error-based tasks [16], whereas those with basal ganglia impairments struggle with reward-based learning [15]. Other studies, but in infants with and without CP, have highlighted the benefits of combining error-based and reward-based learning to enhance skill acquisition and retention [7]. However, the literature in adults with CP, particularly for those with more severe profiles such as quadriplegics, remain sparce. Integrating error-based and reward-based learning insights can lead to tailored interventions, enhancing autonomy and quality of life for those with severe impairments [17]. Future research holds the potential for significant advancements in this field.

The primary aims of this study are: i) to explore the behaviour of adults with and without severe athetoid and ataxic CP when performing error-based and reward-based tasks; ii) to observe short-term and long-term learning retention, in both types of tasks, in adults with and without CP; and iii) to study learning differences in individuals with CP according to task type (i.e., reward-based and error-based). By addressing these objectives, this research seeks to fill a significant gap in the literature and contribute to a better understanding of how to optimise motor learning interventions for individuals with severe CP.

This study hypothesises that individuals with CP will face greater difficulties in learning both error-based and reward-based tasks in comparison with adults without CP. This hypothesis aligns with previous findings indicating that individuals with cerebellar and basal ganglia dysfunctions display increased movement variability, which intensifies with the severity of the impairment [18]. Specifically, it is anticipated that those with ataxia will exhibit more pronounced challenges in error-based tasks, not only because of their higher motor variability, but especially due to the central role of the cerebellum in error-based learning. Conversely, those with athetosis are expected to show greater difficulties in reward-based tasks, given the involvement of the basal ganglia in reinforcement-based learning.

## Methods

### Participants

Participants were recruited through emails and telephone calls to various centres. The objective of the project and the profile of the users to be recruited were explained to the leads of each centre. Those who agreed to participate received a project information letter, signed by the Principal Investigator (PI) of the project, as well as informed consent forms for the participants. Following the recruitment process, thirteen individuals with quadriplegic CP were enrolled (see Table 1 for demographic details). In total, 29 individuals were initially screened for eligibility. Of these, 16 were excluded (8 due to spastic CP, 5 due to associated intellectual disability, and 3 due to severe visual impairments). The final sample included 13 participants with ataxic or athetoid CP.

**Table 1:** Demographics details of participants with Cerebral Palsy.

Participants	Diagnosis	Aged (y)	Sex	Weight (kg)	Height (cm)	GMFCS	MACS
		M ± SD		M ± SD	M ± SD	(level)	(level)
		38.18 ± 10.36		61.82 ± 5.62	164.5 ± 6.61		
S1	Athetosis	34	M	58	163	IV	IV
S2	Athetosis	29	F	56	152	IV	IV
S3	Ataxia	45	M	66	155	III	III
S4	Athetosis	54	F	61	166	IV	IV
S5	Ataxia	35	M	67.3	168	IV	III
S6	Athetosis	23	M	60	165	III	III
S7	Athetosis	28	M	65	170	IV	IV
S8	Athetosis	32	M	71	176	III	III
S9	Athetosis	42	M	62	165	III	III
S10	Ataxia	45	M	64	171	III	III
S11	Athetosis	53	F	49,8	158	III	III
S12	Athetosis	23	M	58	166	IV	III
S13	Ataxia	41	F	56.7	161	IV	IV

Y = Years; kg = kilograms; M = Male; F = Female; GMFCS = Gross Motor Function Classification Scale; MACS = Manual Ability Classification Scale.

Participants exhibited severe physical impairments and were classified at level III or IV of the Gross Motor Function Classification System (GMFCS) [19] and level III or IV in the Manual Ability Classification System (MACS) [20] (See Table 1). Participants were recruited from seven care centres in the Valencian and Andalusian regions of Spain serving individuals with CP or related neurological conditions, which together provide services to approximately 200 adults with CP. All participants met the following inclusion criteria: (i) having a medical diagnosis of athetoid or ataxic CP; (ii) being able to follow the test instructions given by the researchers. The exclusion criteria were as follows: (i) individuals with spastic profile of CP; (ii) those with intellectual impairments; (iii) visual deficits that prevented them from correctly seeing the visual information provided by a computer screen (screen size = 27”).

Additionally, a control group (CG) of 18 adults without any physical impairments was included in the study (see Table 2 for demographic details). Ethical approval was obtained from the local University Ethics Committee (Ref. DPS.FMH.01.16). All participants provided their written informed consent prior to data collection.

**Table 2:** Demographics details of control group participants.

Participants	Aged (y)	Sex	Weight (kg)	Height (cm)
	M ± SD		M ± SD	M ± SD
	36.2 ± 12.1		70.4 ± 12.3	172.6 ± 9.1
C01	32	F	63.5	167
C02	28	M	84	186
C03	28	F	64	167
C04	26	M	73.5	178
C05	47	M	79.5	179
C06	29	M	90	170
C07	36	F	64	170
C08	59	F	47	160
C09	32	M	78	180
C10	27	M	70	179
C11	45	M	81	168
C12	41	M	80	185
C13	23	F	62	179
C14	59	F	55	163
C15	29	M	73	170
C16	28	F	49	151
C17	57	M	84	180
C18	26	M	69	176

Y = Years; kg = kilograms; M = Male; F = Female

### Instruments and procedure

A cross-sectional design was performed where participants performed a computer task a vertical fixed handle attached to a dynamometer, functioning as a joystick (FSSB-R3 Warhog, RealSimulator, Madrid, Spain), to move a virtual square following a target. The dynamometer was a two-axes force sensor (measuring medial-lateral and anterior-posterior forces, 98 mm, 60 mm; 350 g) with a maximum sensitivity of 0.0001221 lb and a maximum allowable force of 8 lb. This device included an automatic calibration that was initiated before each recording. It was connected to the computer via USB, and the sample rate was set at 100 Hz [8].

**Fig 1:**
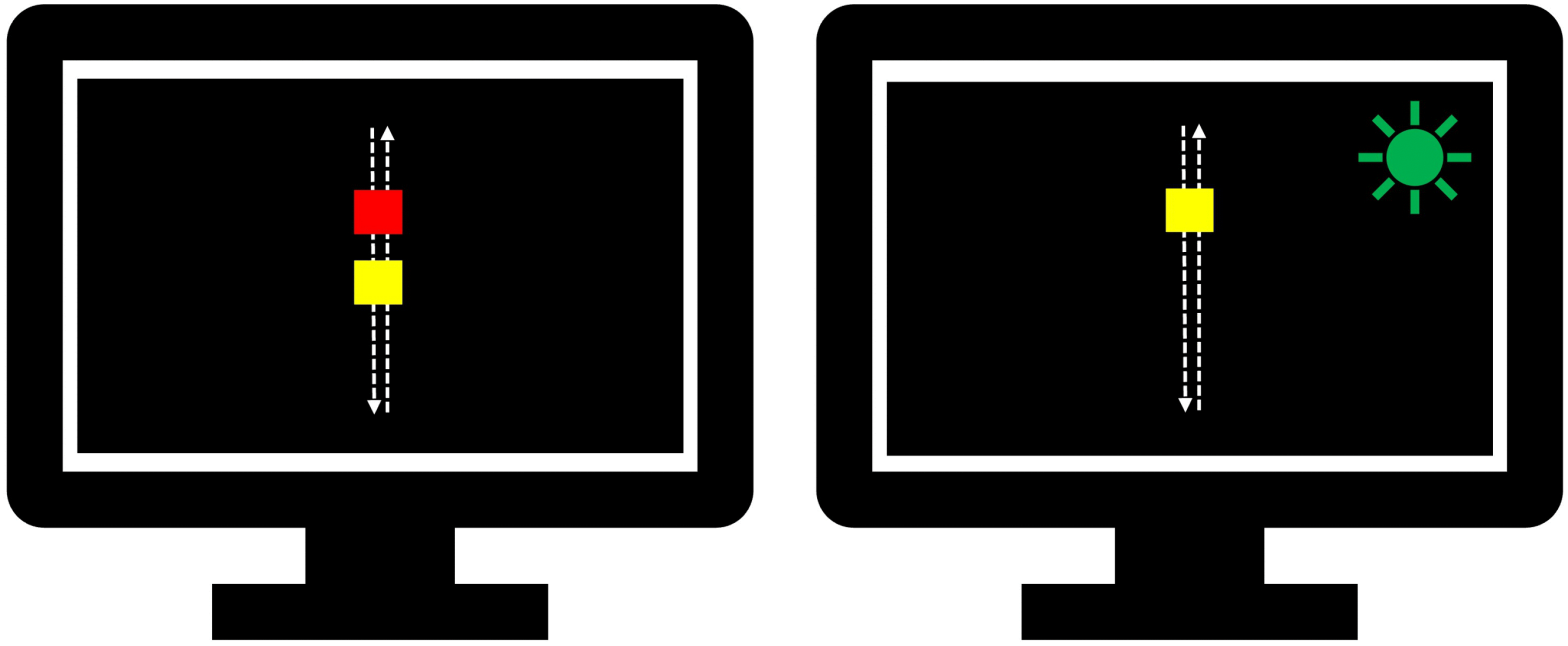
Participant position and joystick grip patterns and visualization of tasks. The left panel illustrates the error-based condition, whereas the right panel illustrates the reward-based condition. The yellow square represents the target to be followed by participants, whereas the red square represents their joystick movement. The red square was visible only in the error-based task.

Participants were seated in their wheelchairs in front of a height-adjustable table, with a screen displaying the task and the joystick placed on the proximal edge of the table. The task was performed using the less affected arm, which was positioned in alignment with the shoulder, hand, and joystick. The arm not involved in the task was positioned across the midline of the body to reduce the degrees of freedom that could interfere with the task. Participants were instructed to hold the joystick in a manner that allowed them optimal control (see Fig 1).

Two continuous tasks were conducted: (i) error-based and (ii) reward-based tasks, specifically designed by the researchers for this study using LabVIEW Software v.11 (National Instruments, Austin, TX, USA).

In the error-based task, a red square is projected on the screen, which takes a trajectory in a straight line, in an upward and downward direction for 45 s at a velocity of 0.020 Hz. The joystick movement for the participant was projected as a yellow square on the screen (same size). Therefore, the purpose of the task was to follow the trajectory of the red square as closely as possible, being aware of how close or far the target was through continuous feedback on task performance.

In the reward-based task, participants tracked the target (a red square) moving along a previously described vertical trajectory, although this movement was not displayed on the screen. Therefore, no constant feedback was provided, and only when the yellow square was in contact with the target square, a green light on the right frame of the monitor lighted up, indicating to the participant that the tracking of the target was correct. The permissible error to hit the target was 10% of the maximum force range exerted to cover the target's trajectory. This maximum force was consistently set for all participants at 1.80 lb.

**Fig 2:**
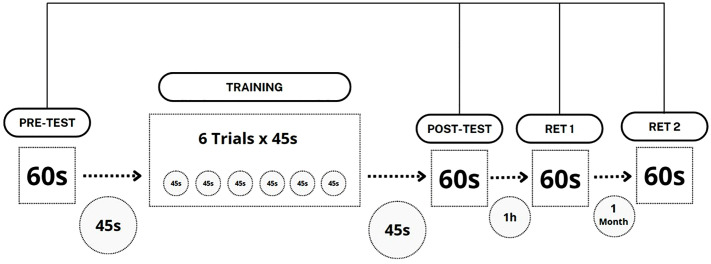
Assessment protocol.

To assess learning retention, participants were assessed twice with a month of difference between them. Subjects were divided into two groups, and training was counterbalanced by task type so that half started with the error task and half with the reward task. The following month, each group started with the task they had not previously performed. The protocol consisted of an initial 10-second calibration of the equipment and software. The first performance measurement began with a pre-test, which had a duration of 60s with 45s of rest. Then 6 training trials were performed with a duration of 45s each and a rest of 45s between them. Subsequently, three more performance measurements were applied: the post-test, re-test one (RET1), and re-test two (RET2). The first re-test is carried out one hour after the post-test, and finally, the second re-test is carried out after one month (see Fig 2).

### Data analysis and reduction

The application, designed using LabVIEW Software v.11, recorded the time series of the relative and absolute error, measured in Newtons (N), on the vertical axis at 100 Hz. Through this application, the time series were also subsampled at 50 Hz, and a low-pass filter (4th-order, zero-phase-lag, Butterworth, 20 Hz cut-off frequency) was applied.

After these procedures, two different time series were obtained. First, the absolute error (AE) was calculated as the magnitude of the error, taking the absolute values of the relative error, and its average was used to assess participants’ accuracy. Second, the relative error (RE) was defined as the distance between the target and the square controlled by the participant, capturing the positive or negative fluctuations of the participant's performance relative to the target. The RE time series was utilised to assess the participants’ variability, with its standard deviation measuring the amount of variability and long-term Detrended Fluctuation Analysis (DFA) evaluating the variability structure. The structure of variability was computed by the DFA (for computation details, see Peng et al. (1995) using another application designed using LabVIEW Software v.11.

To measure the effect of practice, the relative learning ratios (LR) were computed by comparing the AE during the pre-test (AE_PRE_) with the tests performed after the practice period: post-test (LR_PRE-POST_), and the two retention tests (LR_PRE-RET1_ and LR_PRE-RET2_). The equations used were as follows:

LR_PRE-POST_ = $\left[\text{100}*\left({AE}_{PRE}-{AE}_{POST}\right)/{AE}_{PRE}\right]$; LR_PRE-RET1_ = $\left[\text{100}*\left({AE}_{PRE}-{AE}_{RET\text{1}}\right)/{AE}_{PRE}\right]$; LR_PRE-RET2_ = $\left[\text{100}*\left({AE}_{PRE}-{AE}_{RET\text{2}}\right)/{AE}_{PRE}\right]$.

To compute the average of the AE, the standard deviation of the relative error, and the different learning ratios, the Microsoft Excel application was used. This non-linear tool is based on a modification of a classic root mean square analysis of the random walk, which evaluates the presence of long-term correlations within the time series by a parameter referred to as the scaling index (α) [21,22]. Different values of α indicate the following: α > 0.5 implies persistence (i.e., the trajectory tends to continue in its current direction); α < 0.5 implies antipersistence (i.e., the trajectory tends to return to where it came from); and α = 0.5 implies an uncorrelated signal [22]. Therefore, α identifies the extent to which further data are dependent on the previous [23]. In previous studies, DFA has been used to assess motion adjustments [1,24]. To maximize the long-range correlations and to reduce the estimation error of α, long-term correlation was characterized by the slope α obtained from the range of 4 ≤ n ≤ N/10, where n is the box size, and N is the data length [25].

### Statistical analysis

Normality of the variables was evaluated using the Shapiro-Wilk test due to the sample size. Most variables did not follow a normal distribution; thus, nonparametric tests were used for all analyses. First, to evaluate the influence of initial performance on learning progression, Spearman’s rank correlation coefficients (ρ) were calculated between AE_PRE_ scores and the different learning rates (LR_PRE-POST_, LR_PRE-RET1_, LR_PRE-RET2_). In some cases, the initial level of performance was related to the learning rate, particularly in the CP group (see Table 4). Thus, to avoid initial performance bias in the learning rate, linear regression analyses were carried out for each group comparing the AE_PRE_ with the three different learning rates (LR_PRE-POST_, LR_PRE-RET1_, LR_PRE-RET2_). To obtain those residual scores, the procedure used in previous studies [1,8,26] was followed: i.e., a linear regression was estimated between initial performance (AE_PRE_) and the learning ratios (LR). For the correlational analysis, the participants’ residual scores were used to compute the residual learning index (RLR) using the equations described in the Data Analysis and Reduction subsections, rather than the original AE values.

Statistical comparison analyses were conducted separately for each group (CG and CP) and task type (reward and error-based). First, Friedman tests were employed to assess changes in performance (AE) and variability structure (DFA) across the four evaluation tests (Pre, Post, Retest 1, and Retest 2), with Conover’s post hoc comparisons used to identify specific differences between evaluation pairs. Then, to identify differences between task types and between groups at specific evaluation tests, the Wilcoxon signed-rank test and the Mann-Whitney U test were used to account for the related nature of the samples, respectively. To evaluate interaction effects (*Group × Evaluations or Task Type × Evaluations*), learning rates (LR) were compared using Mann-Whitney U tests; a significant result indicated that the rate of change over time differed between conditions. Additionally, Spearman’s rank correlation coefficients (ρ) were calculated to examine the relationships between initial performance (AE_PRE_), learning rates (RLR_PRE-POST_, RLR_PRE-RET1_, RLR_PRE-RET2_), and variability structure (DFA_PRE_).

Finally, a subgroup analysis was performed within the CP group based on diagnosis: Athetosis (AT) and Ataxia (AX). Following the same non-parametric protocol, Friedman tests assessed changes over time within each subgroup, while the Wilcoxon signed-rank and Mann-Whitney U tests were used to compared differences between task types and between groups (AT and AX) across evaluation tests. All statistical analyses were performed using JASP (version 0.19.3), with a significance level of *p* < 0.05.

## Results

Descriptive statistics for each group are presented in Table 3. Overall, values decreased from pre-test to post-test across both groups and tasks. However, the CP group consistently showed higher values than the CG.

**Table 3:** Average and standard deviation of the performance (AE) and the structure of variability (DFA).

		PRE-TEST	POST-TEST	RE-TEST 1	RE-TEST 2
REWARD-BASED TASK		CONTROL GROUP			
	AE	9.06 ± 7.04	4.59 ± 2.97	4.25 ± 2.72	4.50 ± 3.32
	DFA	1.09 ± 0.23	0.98 ± 0.23	1.02 ± 0.27	1.05 ± 0.27
		CEREBRAL PALSY GROUP			
	AE	14.53 ± 5.39	13.05 ± 5.58	12.88 ± 6.23	13.57 ± 6.05
	DFA	1.24 ± 0.21	1.15 ± 0.29	1.15 ± 0.26	1.11 ± 0.34
ERROR-BASED TASK		CONTROL GROUP			
	AE	0.38 ± 0.19	0.26 ± 0.11	0.25 ± 0.09	0.27 ± 0.11
	DFA	0.76 ± 0.11	0.75 ± 0.13	0.78 ± 0.14	0.79 ± 0.17
		CEREBRAL PALSY GROUP			
	AE	4.26 ± 4.87	2.72 ± 3.66	2.68 ± 3.20	2.79 ± 2.66
	DFA	0.94 ± 0.33	0.90 ± 0.30	0.99 ± 0.26	0.95 ± 0.24

AE = absolute error; DFA = Detrended Fluctuation Analysis.

Table 4 displayed the results from Spearman’s rank correlation coefficients between AE_PRE_ and the different learning rates (LR_PRE-POST_, LR_PRE-RET1_, LR_PRE-RET2_), showing how the initial performance influences the learning rate. Thus, to avoid the initial performance bias in the learning rate, residual learning rates (RLR) were used for the remainder of the correlational analysis, as mentioned in the Statistical Analysis subsection.

**Table 4:** Correlations between learning rates and the initial values of performance (AE_PRE_).

CONTROL GROUP	REWARD-BASED TASK	ERROR-BASED TASK
	AE_PRE_	AE_PRE_
LR_PRE-POST_	0.135	0.550*
LR_PRE-RET1_	0.501*	0.447*
LR_PRE-RET2_	0.323	0.112
CEREBRAL PALSY	REWARD-BASED TASK	ERROR-BASED TASK
	AE_PRE_	AE_PRE_
LR_PRE-POST_	0.497*	0.308
LR_PRE-RET1_	0.443*	0.374*
LR_PRE-RET2_	0.610*	0.388

AE_PRE_ = absolute error displayed in the pre-test; LR = learning rate; PRE-POST = differences in the absolute error between the pre-test and post-test; PRE-RET1 = differences in the absolute error between the pre-test and re-test 1; PRE-RET2 = differences in the absolute error between the pre-test and re-test 2. * *p* < 0.05.

**Fig 3:**
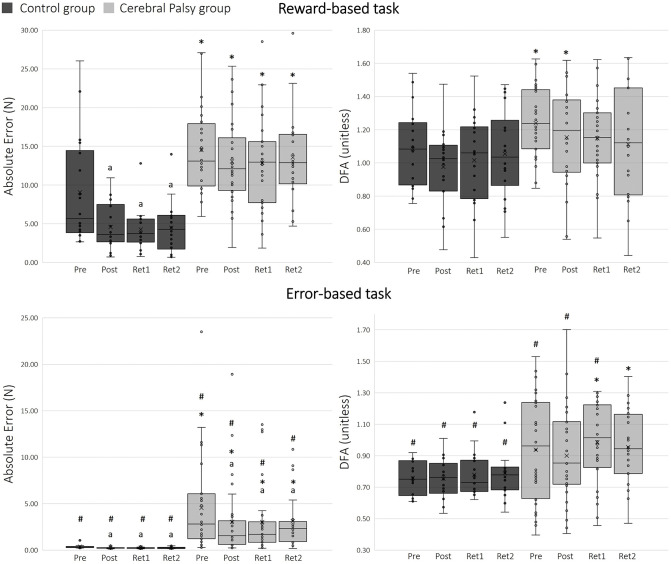
Significant differences between tests and groups (CG = control group; CP = cerebral palsy group) in the reward-based (top of the figure) and error-based task (bottom of the figure) for performance (AE) and variability structure (DFA). DFA = Detrended Fluctuation Analysis; PRE = pre-test; POST = post-test; RET1 = re-test 1 (after 1-h rest); RET2 = re-test 2 (after 1-month rest). a = significant differences compared with the pre-test. * = significant differences between groups. *p* < 0.05. # = significant differences between tasks.

Concerning the progression after the training session, different tendencies were found depending on the type of learning task (Fig 3). In the reward-based task, the CG showed a significant improvement (*p* < 0.01), displaying significantly lower AE in the post-test, re-test 1, and re-test 2 compared to the pre-test. However, this progression was not found in the groups with CP. In addition, these differences were not related to changes in the variability structure for any of the groups. On the other hand, in the error-based task, both groups showed a significantly improvement (CG: *p* < 0.01; CP: *p* = 0.047), with lower AE values in the post-test (CG: *p* < 0.01; CP: *p* = 0.009), re-test 1 (CG: *p* < 0.01, CP: *p* = 0.023), and re-test 2 (CG: *p* < 0.01, CP: *p* = 0.053) than in the pre-test. Nevertheless, no differences were found in the variability structure (DFA values).

The analysis comparing the differences between the type of task showed that both groups displayed significantly better performance (lower AE) (*p* ≤ 0.01) and a higher number of adjustments (lower DFA) (CG: *p* < 0.01; CP: *p* < 0.05) in the error-based task than in the reward-based task. The only situation in which these differences were not found was in the DFA values displayed in retest 2 for the CP group (*p* = 0.058) (Fig 3).

Regarding between-group differences, participants in the CG consistently showed typical performance, with lower AE values, whereas the CP group exhibited higher error rates across all evaluation tests in both tasks (see Fig 3). In addition, CG participants also displayed lower DFA values than the CP group. In the reward-based task, differences in DFA were observed at pre-test and post-test, but were no longer present at the retests. Conversely, in the error-based task, differences emerged from Retest 1 onward.

**Fig 4:**
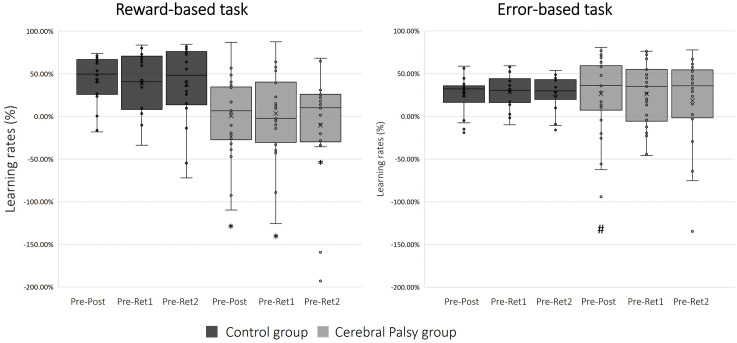
Significant differences between tests and groups (CG = control group; CP = cerebral palsy group) in the reward-based (left-side of the figure) and error-based task (right-side of the figure). PRE = pre-test; POST = post-test; RET1 = re-test 1 (after 1-h rest); RET2 = re-test 2 (after 1-month rest). * = significant differences between groups. *p* < 0.05. # = significant differences between tasks.

As mentioned above, to assess the interaction effects, learning rates (LR) were compared (Fig 4). Differences were found between groups only in the reward-based tasks. The CG learned significantly more than the CP group (LR_PRE-POST_: *p* < 0.01; LR_PRE-RET1_: *p* = 0.013; LR_PRE-RET2_: *p* = 0.006). On the other hand, no significant differences were found between the type of task in the CG according to participants’ LR, but the CP group learned significantly more in the error-based task than in the reward-based task (LR_PRE-POST_: *p* = 0.010), although these differences disappeared in the re-tests (LR_PRE-RET1_: *p* = 0.069; LR_PRE-RET2_: *p* = 0.144).

Once the differences between groups were assessed, correlation analyses were performed to check if the variability structure was related to performance and/or learning rate. As shown in Table 5, these correlations varied according to task type and group. Regarding the relationship between initial performance and variability structure, no correlations were observed in the CG for either the reward-based or the error-based task. In the CP group, no correlation was found in the reward-based task; however, a correlation was observed in the error-based task. Specifically, higher AE values were associated with higher DFA values during the pre-test.

**Table 5:** Correlations between normalized learning rates and the initial values of performance (AE_PRE_) and variability structure (DFA_PRE_).

CONTROL GROUP	REWARD-BASED TASK		ERROR-BASED TASK	
	AE_PRE_	DFA_PRE_	AE_PRE_	DFA_PRE_
DFA_PRE_	0.160	--	0.404	--
RLR_PRE-POST_	−0.183	−0.241	0.042	0.209
RLR_PRE-RET1_	−0.305	−0.028	0.033	−0.099
RLR_PRE-RET2_	−0.236	−0.007	−0.402	−0.061
**CEREBRAL PALSY**	**REWARD-BASED TASK**		**ERROR-BASED TASK**	
	AE_PRE_	DFA_PRE_	AE_PRE_	DFA_PRE_
DFA_PRE_	0.102	--	0.518**	--
RLR_PRE-POST_	0.104	−0.125	0.454*	−0.441*
RLR_PRE-RET1_	0.128	−0.322	−0.160	−0.014
RLR_PRE-RET2_	0.196	−0.284	−0.356	−0.102

AE_PRE_ = absolute error displayed in the pre-test; DFA_PRE_ = Detrended Fluctuation Analysis displayed in the pre-test; RLR = residual learning rate; PRE-POST = differences in the absolute error between the pre-test and post-test; PRE-RET1 = differences in the absolute error between the pre-test and re-test 1; PRE-RET2 = differences in the absolute error between the pre-test and re-test 2. *p < 0.05; **p < 0.01.

Note: Positive correlations indicate that higher DFA values were associated with higher AE values. *p < 0.05; **p < 0.01.

Regarding the relationship between the learning rate and variability structure, only one significant correlation was found for the group with CP between the learning rate computed comparing the pre-test and post-test and the initial DFA. Participants with CP who initially displayed a higher number of adjustments (lower DFA) displayed a higher learning rate during the error-based task, but these participants were also the ones with lower performance (i.e., higher AE) (Table 5).

Additional analyses were carried out considering the diagnosis of the CP group (athetoid or ataxic CP). Descriptive statistics for both CP groups are presented in Table 6.

**Table 6:** Average and standard deviation of the performance (AE) and the structure of variability (DFA) for the CP group according to the diagnostic.

		PRE-TEST	POST-TEST	RE-TEST 1	RE-TEST 2
REWARD-BASED TASK		ATHETOID CP SUBGROUP			
	AE	14.29 ± 6.07	13.64 ± 5.89	13.31 ± 6.80	14.48 ± 6.80
	DFA	1.25 ± 0.20	1.14 ± 0.32	1.13 ± 0.29	1.08 ± 0.38
		ATAXIC CP SUBGROUP			
	AE	15.12 ± 3.35	11.45 ± 4.63	11.84 ± 4.83	11.74 ± 4.08
	DFA	1.20 ± 0.27	1.19 ± 0.20	1.19 ± 0.16	1.18 ± 0.28
ERROR-BASED TASK		ATHETOID CP SUBGROUP			
	AE	4.08 ± 5.43	2.83 ± 4.11	2.63 ± 3.54	2.88 ± 2.75
	DFA	0.88 ± 0.33	0.89 ± 0.32	0.97 ± 0.27	0.93 ± 0.26
		ATAXIC CP SUBGROUP			
	AE	4.57 ± 3.09	1.97 ± 1.32	2.31 ± 1.46	1.94 ± 1.10
	DFA	1.09 ± 0.34	0.93 ± 0.28	0.97 ± 0.25	0.95 ± 0.22

CP = cerebral palsy; AE = absolute error; DFA = Detrended Fluctuation Analysis.

**Fig 5:**
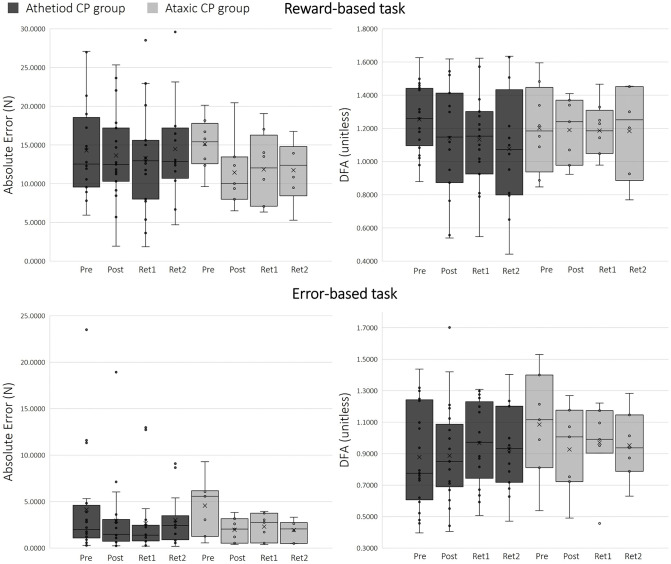
Significant differences between tests and diagnostic subgroups in the reward-based (top of the figure) and error-based (bottom of the figure) tasks for performance (Absolute Error) and variability structure (DFA). DFA = Detrended Fluctuation Analysis; PRE = pre-test; POST = post-test; RET1 = re-test 1 (after 1-h rest); RET2 = re-test 2 (after 1-month rest). a = significant differences compared with the pre-test. * = significant differences between groups. *p* < 0.05. # = significant differences between tasks.

Regarding the initial differences between diagnostic groups (Fig 5), no differences in performance or variability structure were found. Relating to the progression after the training session, in the reward-based task, any group obtained a significant improvement after the training sessions (AT: *p* = 0.475; AX: *p* = 0.218). However, in the error-based task**,** participants with ataxic CP improved after training (*p* = 0.029), displaying lower AE values in the post-test (*p* = 0.007), re-test 1 (*p* = 0.035), and re-test 2 (*p* = 0.004) compared with the pre-test. Concerning the variability structure, no general differences were found (RE: AT *p* = 0.231, AX *p* = 0.516; ER: AT *p* = 0.293, AX *p* = 0.168), but Conover’s post hoc pairs comparisons showed some significant differences. Participants with athetoid CP, in the reward-based task, showed significantly lower DFA values in the re-test 1 (*p* = 0.044) compared with the pre-test, while participants with ataxic CP displayed lower DFA values in the post-test (p = 0.041) than in the pre-test in the error-based task (Fig 5).

Differences between the type of task appeared in both groups. The athetoid CP subgroup clearly showed lower performance (higher AE) and a higher number of adjustments (lower DFA) (*p* < 0.05) in the error-based task than in the reward-based task. Performance differences were also found in the ataxic CP subgroup for the pre-test (*p* = 0.031) and re-test 1 (*p* = 0.031), while no differences were found for DFA values. And regarding the differences between learning rates (LR), to assess the interaction effects, no differences were found between groups in any tasks.

## Discussion

The primary aims of this study are to explore the behaviour of adults with and without severe athetoid and ataxic CP when performing error-based and reward-based tasks, to observe both short-term and long-term learning retention, and to study learning differences in individuals with CP according to both types of tasks. By addressing these objectives, this research seeks to fill a significant gap in the literature and contribute to a better understanding of motor learning interventions for individuals with severe CP.

Motor variability would impact the learning capabilities of individuals with CP. They exhibit greater DFA values when performing tasks compared to those without disabilities (i.e., pre- and post-test assessments in the reward-based task, and re-test 1 and 2 assessments in the error-based task), leading to higher absolute error (AE) and a lower capacity for adjustments in both learning tasks than CG. The study of the structure of variability, such as through DFA, provides complementary information to the magnitude of variability. While the magnitude of variability indicates the extent of performance fluctuations, the structure reveals how these fluctuations are organized over time. In individuals with CP, the higher DFA values suggest a more predictable and less adaptable motor system, which is less responsive to immediate feedback and, therefore, less capable of fine-tuning motor actions through error correction [1]. This relationship suggests that individuals with CP may have difficulty adapting their motor responses effectively, resulting in compromised learning outcomes. The anticipated role of variability in reward-based tasks—fostering exploration and adaptation by encouraging a broader search of movement strategies [27] —was not observed in individuals with CP in this study. For individuals with CP, the benefits of this exploratory variability may be less pronounced due to inherent motor control issues, which can limit the effective implementation of these strategies. Moreover, the elevated DFA values observed in individuals with CP suggest that the inherent motor stability and precision deficits in CP further complicate the motor learning process for this population [28].

These results are consistent with the observed performance patterns, indicating that error-based tasks facilitated learning in the CP group, whereas reward-based tasks did not show comparable improvements. However, this effect was primarily observed in the pre–post comparisons. Therefore, error-based tasks may be relatively more accessible for individuals with CP than reward-based tasks under the conditions examined in this study. In error-based tasks, the CP group showed significant improvement in performance (decreased AE) in post-training tests, whereas no such improvement was observed in reward-based tasks, indicating a floor effect. This suggests that tasks involving error correction enable this group to learn more effectively. This aligns with Schoenmaker et al. [29] who proposed that external feedback positively contributes to meeting task requirements within this collective. Furthermore, Cashaback et al. [30] suggest that when error and reinforcement feedback are in conflict, the sensorimotor system places greater emphasis on error feedback over reinforcement feedback.

However, significant differences in learning behaviours were observed when comparing individuals with cerebellar-origin CP and those with basal ganglia-origin CP. Our study hypothesized that individuals with ataxic CP would exhibit more pronounced challenges in error-based tasks due to their higher motor variability and possibly because of the implications of the cerebellum in this type of learning [10]. This hypothesis aligns with previous findings indicating that individuals with cerebellar and basal ganglia dysfunctions display increased movement variability, which intensifies with the severity of the impairment [18]. Moreover, individuals with ataxic CP, despite the small sample size in this study, seemed to show better conditions for adapting to error-based tasks. The authors suggest that this may be explained, first, by the possibility that these individuals exhibit fewer involuntary movements and reduced degrees of freedom, allowing better task adjustment; and second, by a plausible influence of the sensory component of ataxia. However, it is important to note that only two participants in our group presented with a pure ataxic profile, with the rest having mixed features, which likely impacts the results that can be drawn from this subgroup. Nevertheless, we believe that this study contributes to the literature by offering a more specific understanding of how different forms of CP affect motor learning and adaptability to various tasks.

Learning and long-term retention of motor skills are essential for improving functional abilities in individuals with CP. Previous research indicates that although individuals with CP can acquire new motor tasks, they often require more repetitions and extended practice periods to reach levels of proficiency comparable to those without disabilities [31]. In line with this, our findings suggest that when task difficulty is appropriately adjusted—as in the error-based task used in this study—individuals with CP can retain performance improvements. However, sustaining these gains over longer periods may require continued practice and reinforcement. From a clinical perspective, these results support the use of training approaches that provide clear and continuous visual feedback about movement errors, such as visually guided reaching or tracking tasks. Furthermore, assessing movement variability structure (e.g., DFA) may help clinicians tailor task difficulty and select functional activities—such as reaching, object manipulation, or assistive device control—that better align with each individual’s learning capacity.

Finally, another contribution of this study concerns the target population. Individuals with severe profiles, classified as levels III and IV of the GMFCS [32], and individuals with athetosis and ataxia profiles are less commonly included in studies due to the complexity of recruiting the sample and the potential adaptations and support they may require during assessment, given the severity of their condition. Most previous studies have focused on spastic profiles, leaving a gap in the literature on other forms of CP [33,34].

## Conclusions

The findings of this study underscore the importance of personalised approaches in motor learning interventions for individuals with CP. We have found that individuals with CP exhibited an increased error and a reduced capacity for adjustments. This indicates that CP affects motor stability and precision, leading to a more predictable yet less adaptable motor system, which complicates effective error correction and overall motor learning. The study addressed a gap by including individuals with severe CP profiles (GMFCS III and IV), providing insights into motor learning and adaptability across different CP forms. CP individuals can retain improvements in adjusted tasks but may need ongoing practice for long-term retention. These results provide insights into motor learning in adults with athetoid and ataxic CP, highlighting the need for tailored interventions for these subtypes. For example, in a reaching or tracking task, instead of providing continuous error-correction feedback (e.g., detailed information about trajectory deviation), clinicians might initially implement structured practice with clear success criteria and reward-based feedback (e.g., points, visual reinforcement, or goal attainment signals) when the target is reached within an acceptable range. This approach may help stabilise performance and promote engagement before progressively introducing more precise error-correction strategies as motor control improves.

By understanding the specific challenges and strengths associated with different CP subtypes, practitioners can design more effective rehabilitation and education programmes. Future research should continue to explore the nuances of motor learning in CP to develop targeted interventions that optimise outcomes for this diverse population.

## Limitations

This study has several limitations that should be acknowledged. First, the sample size was small, particularly within the ataxic and athetoid subgroups, which may limit the generalisability of the findings. In addition, the heterogeneity of CP profiles may have introduced variability not fully captured in the analyses. The follow-up period was also limited to one-month, restricting conclusions regarding long-term retention. Finally, the complexity of some tasks, especially the reward task, appeared to challenge participants’ motivation to persist. As a result, some individuals declined to repeat the task at the one-month follow-up, which contributed to sample attrition. Also, the protocols were previously applied to people with no neurological deficit and adapted to the participants’ characteristics (e.g., slightly shorter trial duration and larger target size); they were not validated with this specific sample type before the study. This lack of prior validation with individuals with CP represents a significant limitation that may affect the accuracy of the results, and it should be taken into consideration in future research. Additionally, cognitive status and musculoskeletal restrictions were not formally assessed at recruitment. These factors may have influenced task performance and should be considered in future studies.

Another limitation is that not all participants with CP completed both types of tasks. This was primarily due to individual ability levels, in which certain participants could not sustain performance in one of the tasks, as well as occasional missing data related to fatigue or technical difficulties. These omissions were not systematically linked to a specific diagnosis (ataxic vs athetoid profile) but rather occurred randomly across individuals. Nevertheless, this uneven distribution may have influenced the statistical power of subgroup analyses and should be taken into account when interpreting the results.
