# Correction: Exploring motor variability in adults with severe athetoid or ataxic cerebral palsy: Impact on error-based and reward-based learning

**DOI:** 10.1371/journal.pone.0355274

**Published:** 2026-07-31

**Authors:** Alba Roldan, María Isabel Cornejo, Francisco J. Moreno, Raul Reina, Carla Caballero

The Funding statement for this article is incorrect. The correct Funding statement is as follows: This study was made possible by financial support from the Economy, Industry and Competitiveness Ministry of Spain, Project Cod. DEP2016–79395-P, Spanish Government.
